# Comparative Analysis of the Complete Chloroplast Genomes of Eight *Ficus* Species and Insights into the Phylogenetic Relationships of *Ficus*

**DOI:** 10.3390/life12060848

**Published:** 2022-06-07

**Authors:** Xi Xia, Jingyu Peng, Lin Yang, Xueli Zhao, Anan Duan, Dawei Wang

**Affiliations:** 1 Key Laboratory for Forest Resources Conservation and Utilization in the Southwest Mountains of China, College of Forestry, Southwest Forestry University, Kunming 650224, China; xiaxi@swfu.edu.cn (X.X.); yanglinyl@swfu.edu.cn (L.Y.); zhaoxueli@swfu.edu.cn (X.Z.); duananan@gmail.com (A.D.); 2 Department of Plant Genetics and Breeding, China Agricultural University, Beijing 100089, China; peng.jingyu@outlook.com

**Keywords:** chloroplast genome, *Ficus*, hypervariable regions, phylogenetic relationship

## Abstract

The genus *Ficus* is an evergreen plant, the most numerous species in the family Moraceae, and is often used as a food and pharmacy source. The phylogenetic relationships of the genus *Ficus* have been debated for many years due to the overlapping phenotypic characters and morphological similarities between the genera. In this study, the eight *Ficus* species (*Ficus altissima*, *Ficus auriculata*, *Ficus benjamina*, *Ficus curtipes*, *Ficus* *heteromorpha*, *Ficus* *lyrata*, *Ficus* *microcarpa*, and *Ficus virens*) complete chloroplast (cp) genomes were successfully sequenced and phylogenetic analyses were made with other *Ficus* species. The result showed that the eight *Ficus* cp genomes ranged from 160,333 bp (*F. heteromorpha*) to 160,772 bp (*F. curtipes*), with a typical quadripartite structure. It was found that the eight *Ficus* cp genomes had similar genome structures, containing 127 unique genes. The cp genomes of the eight *Ficus* species contained 89–104 SSR loci, which were dominated by mono-nucleotides repeats. Moreover, we identified eight hypervariable regions (*trnS-GCU*_*trnG-UCC*, *trnT-GGU*_*psbD*, *trnV-UAC*_*trnM-CAU*, *clpP*_*psbB*, *ndhF*_*trnL-UAG*, *trnL-UAG*_*ccsA*, *ndhD*_*psaC*, and *ycf1*). Phylogenetic analyses have shown that the subgenus *Ficus* and subgenus *Synoecia* exhibit close affinities and based on the results, we prefer to merge the subgenus *Synoecia* into the subgenus *Ficus*. At the same time, new insights into the subgeneric classification of the *Ficus* macrophylla were provided. Overall, these results provide useful data for further studies on the molecular identification, phylogeny, species identification and population genetics of speciation in the *Ficus* genus.

## 1. Introduction

The genus *Ficus* is the most numerous species of evergreen plants in the family Moraceae and is mainly found in subtropical and tropical areas [1]. For a long time, numerous *Ficus* species have been utilized as sources for food and pharmacy because of their rich nutritional content [2]. The genus *Ficus* is one of the largest woody genera of angiosperms, and the subdivision of the genus has been a concern of taxonomists [3]. The present relatively complete taxonomic system for the genus *Ficus* was proposed by Berg [4], which divided the genus *Ficus* into six subgenera (subgenus *Urostigma*, subgenus *Pharmacosycea*, subgenus *Sycomorus*, subgenus *Ficus*, subgenus *Sycidium*, and subgenus *Synoecia*) based on previous molecular phylogenetic analyses and morphological characters. However, the use of morphological and molecular markers for the identification of the *Ficus* genus are controversial or limited due to overlapping phenotypic characteristics or morphological similarities among the *Ficus* genus [3,5,6,7]. Therefore, the provision of additionalgenomic information is imperative for the understanding of the *Ficus* genus, and for the safe and effective utilization of *Ficus*.

The chloroplast (cp) is a unique organelle in the plant cell that not only plays a vital part in photosynthesis, but also participates in the synthesis of other organisms [8,9,10]. The cp genomes in plants are uniparentally inherited and consists of a highly conserved genomic structure a small single-copy (SSC), a couple of inverse-repeat (IR) regions, and a large single-copy (LSC), which also has a large number of variable loci [11,12]. Therefore, cp genomes have often been used to research the evolution, interspecific divergence, adaptive population history and phylogeny of related species [12,13,14]. With the emergence of the whole-genome sequencing era, the whole cp genomes have been extensively used in phylogenetic differentiation studies, species taxonomic identification and genetic breeding [15,16,17,18,19]. Although the cp genomes of some genus of *Ficus* have been reported previously [20,21,22,23,24,25,26,27], the eight *Ficus* species studied here have not been reported. As such, we compared the cp genome structures of eight *Ficus* species to provide a useful genomic resource for *Ficus* species.

In our study, the complete cp genomes of eight *Ficus* species were sequenced, which possess economically significant values (*Ficus altissima*, *Ficus auriculata*, *Ficus benjamina*, *Ficus curtipes*, *Ficus heteromorpha*, *Ficus lyrata*, *Ficus microcarpa*, and *Ficus virens*). The structural features and sequence differences of the eight *Ficus* species were then compared and analyzed, while the phylogeny of the eight *Ficus* species was inferred from the reported cp genomes of the *Ficus* genus. The results of this study can provide guidance for the subsequent conservation and utilization of *Ficus* species.

## 2. Materials and Methods

### 2.1. Plant Materials and DNA Extraction

Eight taxa, *F. altissima*, *F. auriculata*, *F. benjamina*, *F. curtipes*, *F. heteromorpha*, *F. lyrata*, *F. microcarpa*, and *F. virens*, represented the genus *Ficus* of the family Moraceae. We collected young, fresh, and non-diseased leaves from the adult plants of the target species, which were then frozen into liquid nitrogen. In addition, the voucher specimens were preserved in the herbarium of Southwest Forestry University, Kunming, Yunnan, China (Table S1). DNA extraction was performed using a modified CTAB method [28].

### 2.2. Genome Sequencing, Assembly and Annotation

Sequencing was performed by Annoroad Gene Technology (Beijing, China) to generate libraries with an average insert size of 400 bp from total DNA on an Illumina-based platform (Illumina Novaseq 6000). Then, the raw data obtained by sequencing were assembled using GetOrganelle v1.6.0 software with *Ficus religiosa* as the reference [29]. The assembled sequences were annotated with Geneious prime using *F. religiosa* as a reference and manually adjusted for start and stop codons. The genome mapping of the annotated *Ficus* cp genomes was conducted using OGdraw online [30]. The GenBank accession numbers of the eight *Ficus* species are shown in Table S1. 

### 2.3. Simple Sequence Repeats (SSR) and Repetitive Sequence Analysis

Identification of SSRs was performed using MISA [31], and the specific parameter settings were as in Zhao et al. [32]. The forward (F), reverse (R), complementary (C), and palindromic (P) oligonucleotide repeats were determined by the REPuter program [33], and then the tandem repeats were subdivided using the network-based Tandem Repeat Finding [34], and the REPuter and Tandem Repeat specific parameters were set as in Yang et al. [35].

### 2.4. Genome Comparison and Divergent Hotspots Identification

Expansion or contraction of the IR region was investigated and visualized by IR scope [36]. The alignment of the eight *Ficus* cp genome sequences of species was performed using MAFFT v7 [37], and the completed sequences were subsequently compared using the Shuffle-LAGAN mode in mVISTA [38]. The nucleotide variability (Pi) of the cp genome was analyzed using DnaSp v5.10 [39], with the following settings: a step size of 200 bp and a window length of 800 bp.

### 2.5. Non-Synonymous (Ka) and Synonymous (Ks) Substitution Rate Analysis

First, the selected protein-coding genes (length > 300 bp) were paired in MAFFT. Then, the Ka/Ks value of the screened genes was calculated using the KaKs_calculator [40] with reference to Ivanova et al. [41].

### 2.6. Phylogenetic Analysis

To estimate phylogenetic relationships of eight *Ficus* species in the genus *Ficus*, cp genomes of 34 taxa were compared (Table S2), including 31 *Ficus* species, and 3 Flacourtiaceae species (*Flacourtia indica*, *Homalium ceylanicum* and *Poliothyrsis sinensis*) were set as outgroups. The sequence alignment of 34 cp genomic matrices was made using MAFFT v7 software. In order to obtain robust phylogenetic relationships, we constructed phylogenetic trees for the genus *Ficus* using the maximum likelihood (ML) method. The optimal nucleotide substitution model (K3Pu + F + R5) was selected using ModelFinder [42]. ML analyses was performed using IQ-tree 1.5.5 [43] under ultrafast bootstrap (1000) and the partition model (partitioned analysis with mixed data) [44]. The ML tree was visualized by using FigTree v1.4.0.

## 3. Results

### 3.1. Genomic Characteristics of Chloroplast 

**Figure 1:**
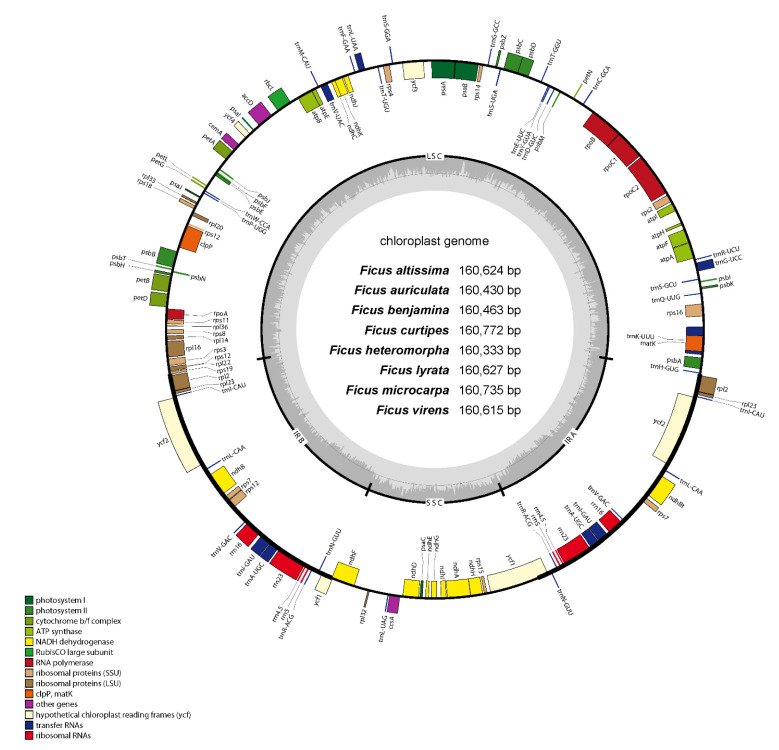
The complete chloroplast genome of eight *Ficus* species. Genes shown outside the circle are transcribed clockwise and those inside counterclockwise. Different colors represent different kinds of functional genes. The dark gray area and light gray area of inner circle represent the ratio of GC content to AT content of the genome respectively.

The cp genomes of *F. altissima*, *F. auriculata*, *F. benjamina, F. curtipes*, *F. heteromorpha*, *F. lyrata*, *F. microcarpa*, and *F. virens* were sequenced, generating approximately 2.61, 2.89, 2.52, 2.35, 2.60, 2.62, 2.52 and 2.56 Gb of paired-end reads, respectively. The average depth of coverage for the eight *Ficus* cp genomes was approximately 3244×.The eight newly sequenced chloroplast genomes of the genus *Ficus* (*F. altissima*, *F. auriculata*, *F. benjamina*, *F. curtipes*, *F. heteromorpha*, *F. lyrata*, *F. microcarpa*, and *F. virens*) were typical quadripartite structure with a length range of 160,333 bp (*F. heteromorpha*) ~ 160,772 bp (*F. curtipes*) (Figure 1). The LSC region of the eight *Ficus* species cp genomes ranged from 88,426(*F. auriculata*) to 89,149 (*F. curtipes*) bp, the SSC region from 19,183 (*F. virens*) to 20,158 (*F. microcarpa*) bp, and the IRb region from 25,743 (*F. curtipes*) to 26,249 (*F. virens*) bp (IRa: 25,743 to 26,249 bp). The cp genomes of all eight *Ficus* were uploaded to NCBI and GenBank accession numbers were obtained (Table S1).

We annotated a total of 127 unique genes, containing 83 protein-coding genes, 8 rRNAs and 36 tRNAs, in eight *Ficus* species showing similar genomic structures. Nineteen of the genes contained one intron and three genes contained two introns (Table 1). In all eight *Ficus* species, their cp genomes were AT-rich, with 64.1% of the genomes made up of A/T nucleotides. The GC content of the IR regions, LSC and SSC regions, protein-coding sequences, tRNA, and rRNA among the eight *Ficus* cp genomes was similar, with 42.4–42.7%, 32.7%, 37.1–37.2%, 52.9% and 55.4% GC content, respectively (Table S3).

### 3.2. SSR and Repetitive Sequence Analysis

**Figure 2:**
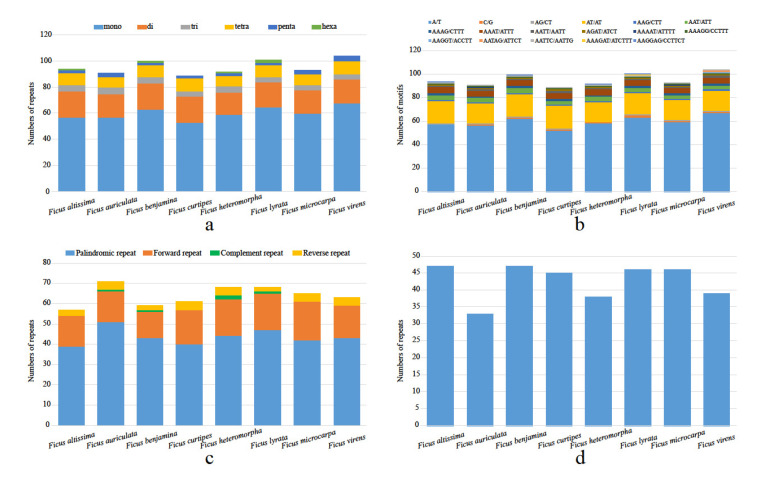
The number and type of simple sequence repeats (SSRs) and repeat sequences in the chloroplast genomes of eight *Ficus* species. (**a**) Frequency of eight SSR types, (**b**) Frequency of SSR motifs in different repeat class types, (**c**) Frequency of five repeat types, (**d**) Frequency of dispersed repeat sequences by length.

We detected 94, 91, 100, 89, 92, 101, 93 and 104 SSR loci in *F. altissima*, *F. auriculata*, *F. benjamina*, *F. curtipes*, *F. heteromorpha*, *F. lyrata*, *F. microcarpa* and *F. virens* cp genomes, respectively. Among them, the richest repeat type was mono-nucleotides (53–68), followed by di-nucleotides (17–20), tetra-nucleotides (8–10), tri-nucleotides (4–5), penta-nucleotides (2–4), and hexa-nucleotides (0–2), respectively. The A/T repeat motif accounted for a large proportion (52–67) among all of the repeat types, followed by AT/AT (17–19), but the other repeat motifs were very rare. Mono-nucleotide repeat motifs (A/T), di-nucleotide repeat motifs (AT/TA), tri-nucleotide repeat motifs (AAG/CTT, AAT/ATT), tetra-nucleotide repeat motifs (AAAG/CTTT, AAAT/ATTT, AATT/AATT, AGAT/ATCT) and penta-nucleotides repeat motifs (AAAGG/CCTTT, AATTC/AATTG) were present in each cp genome (Figure 2a,b; Table S4).

The analysis of the eight cp genomes using REPuter showed that a total of 512 repeats were identified. The number of repeats in each species ranged from 57 (*F. altissima*) to 71 (*F. auriculata*), with palindromic repeats being the most abundant types (39–51), followed by forward repeats (13–19) and reverse (2–4) repeats. The complement repeats are rare in these 8 cp genomes (Figure 2c; Table S5). The results of the tandem repeats showed a total number of 341 tandem repeats for the eight cp genomes, ranging from 33 (*F. auriculata*) to 47 (*F. altissima* and *F. benjamina*) (Figure 2d).

### 3.3. IR Constriction and Expansion

**Figure 3:**
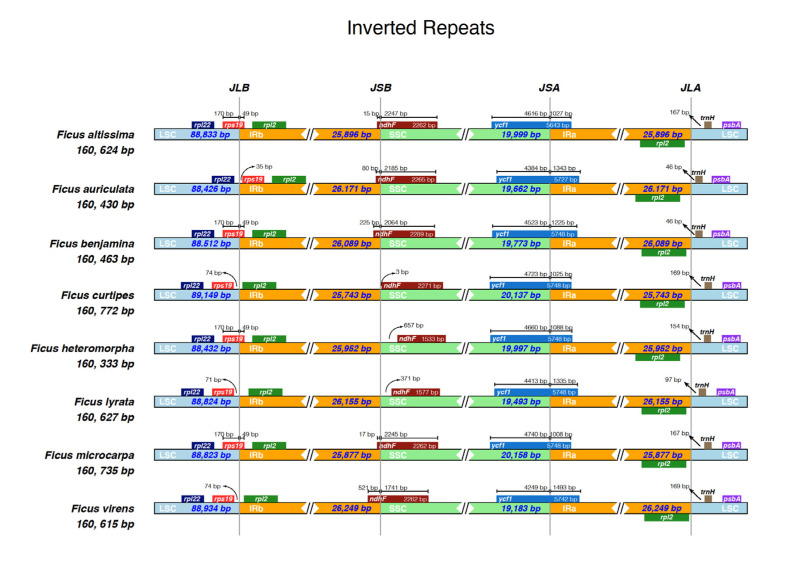
Comparison of the borders of LSC, IR and SSC in the chloroplast genomes of eight *Ficus* species.

We performed a comparative analysis of the contraction and expansion of the IR/SC region boundary in eight *Ficus* species (Figure 3). The genes at the junctions of the eight *Ficus* species included *rps19*, *ndhF*, *rpl22*, *rpl2*, *psbA*, *trnH*, and *ycf1*. The *ndhF* gene of *F. curtipes*, *F. heteromorpha*, and *F. lyrata* was located exclusively in the SSC region, ranging from 3 to 657 bp away from the boundary of JSB. The *ndhF* gene of *F. altissima*, *F. auriculata*, *F. benjamina*, *F. microcarpa,* and *F. virens* was mainly located in the SSC region, but a small proportion of the *ndhF* gene spanned the junction 15 to 521 bp into the IRb region. The rps19 gene of *F. curtipes*, *F. lyrata*, and *F. virens* was located in the LSC region, which for *F. auriculata* was located in the IRb region, while the gene in *F. altissima*, *F. benjamina*, *F. heteromorpha* and *F. microcarpa* spans the LSC and IRb boundary. We also observed that the *ycf1* gene in all eight *Ficus* cp genomes spanned the SSC and IRa junctions, but the majority were located in the SSC region, and the length of the *ycf1* gene in the IRa was within the range of 1008–1493 bp. In the eight *Ficus* cp genomes, the *trnH*, *psbA*, and *rpl22* genes were located exclusively in the LSC region, and the distance between the *trnH* gene and JLA ranged from 46 to 169 bp.

### 3.4. Sequence Divergence Analysis

**Figure 4:**
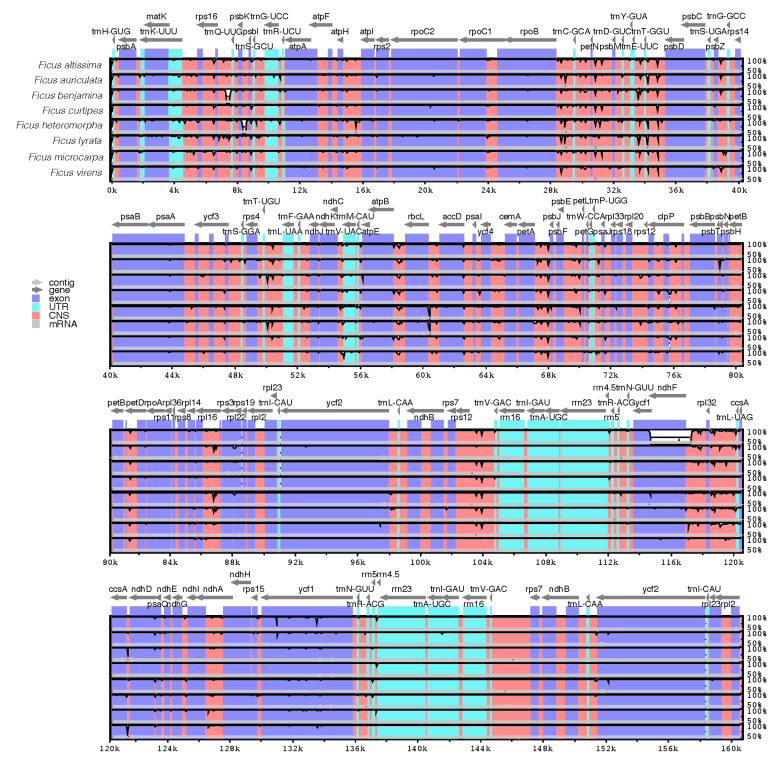
Sequence alignment of the chloroplast genomes of eight *Ficus* species, with *F. religiosa* as a reference. The X-axis represents the coordinates in the chloroplast genome, and the Y-axis represents the percentage of homogeneity, ranging from 50% to 100%.

**Figure 5:**
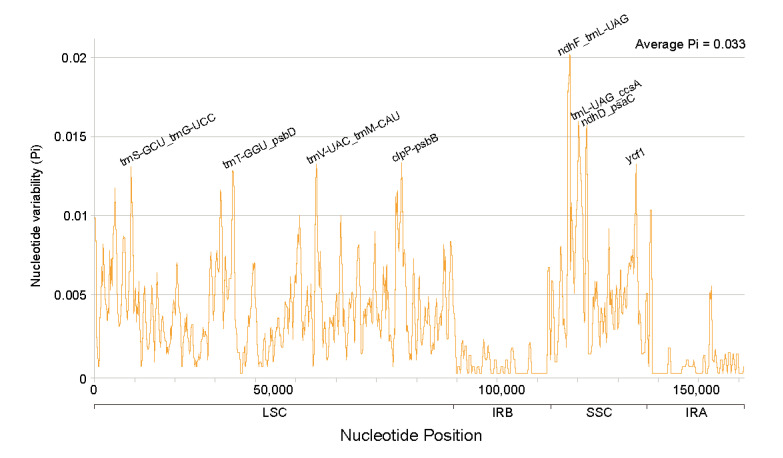
Nucleotide variability (Pi) in the coding region of eight *Ficus* species.

To determine the sequence differences among the eight *Ficus* cp genomes, we used *Ficus religiosa* as a reference genome and compared them using mVISTA (Figure 4). It is apparent from Figure 4 that the eight *Ficus* cp genomes were highly conserved, but regions of divergence among intergenic regions were also found, such as *trnT-UGU*_*trnL-UAA*, *rps16*_*trnQ-UUG*, *trnT-GGU*_*psbD*, *petA*_*psbJ rpoB*_*trnC-GCA*, *petN*_*psbM*, *atpB*_*rbcL*, and *rpl32*_*trnL-UAG*. Subsequently, we calculated the nucleotide variability (Pi), as the Pi value allows for a better assessment of the degree of sequence divergence (Figure 5). From the results, the degree of divergence in the LSC and SSC regions exceeded that of the IR region, and the average value of Pi in the eight *Ficus* species was approximately 0.0033. Then, Pi > 0.0128 was identified as the hotspot region, and a total of eight high variance regions were found: *trnS-GCU*_*trnG-UCC*, *trnT-GGU*_*psbD*, *trnV-UAC*_*trnM-CAU*, *clpP*_*psbB*, *ndhF*_*trnL-UAG*, *trnL-UAG*_*ccsA*, *ndhD*_*psaC*, and *ycf1*, where the *ndhF*_*trnL-UAG* region has the highest Pi value (0.0202). The identification of these highly variable regions offers invaluable information for the evolution of markers in *Ficus*.

### 3.5. Non-Synonymous (Ka) and Synonymous (Ks) Substitution Rate Analysis

**Figure 6:**
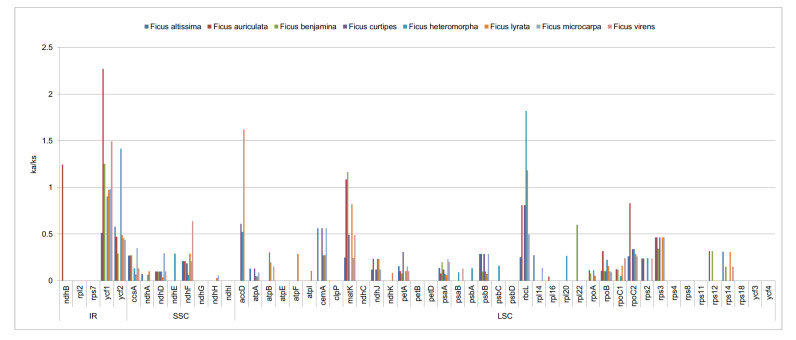
The Ka/Ks ratio of 53 protein-coding genes of eight cp genomes for comparison with *F. religiosa*.

We calculated the Ka/Ks ratios of the eight *Ficus* chloroplast genomes using *F. religiosa* as the reference with a total of 53 protein genes reselected based on 300 bp length (Figure 6; Table S6). The Ka/Ks ratio results indicate that gene-specificity is the majority, and the remaining small portion is region-specific (the IR, SSC, and LSC regions showed comparable values). The Ka range of eight *Ficus* chloroplast genomes was from 0 to 2.6149, and the Ks values ranged from 0 to 5.0893. The highest Ka and Ks was for the *ycf1* gene between *F. altissima* and *F. religiosa* (ka = 2.6149, ks = 5.0893), while the values of Ka and Ks for a large number of genes were equal to 0. The average Ka/Ks ratio for the 53 protein genes was 0.1211 and the average Ka/Ks value for *ycf1* was 1.0476, with only the *ycf1* gene having a Ka/Ks value greater than 1 among the genes tested.

### 3.6. Phylogenomic Analysis

**Figure 7:**
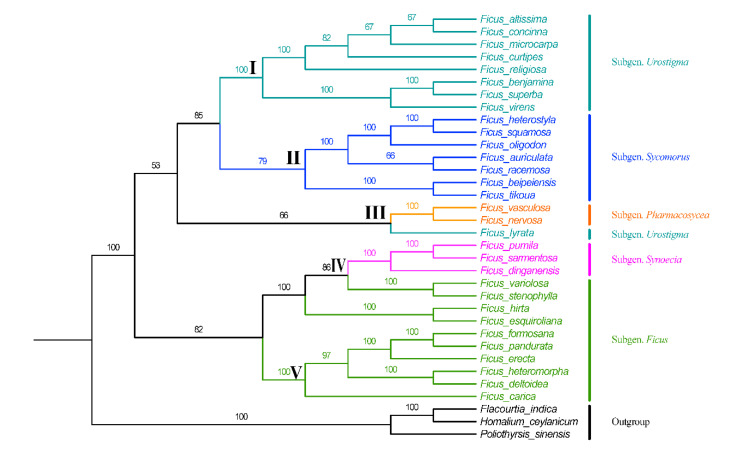
Phylogenetic tree inferred from maximum likelihood (ML) based on the 34 taxa complete chloroplast genomes.

The phylogenetic relationships of the genus *Ficus* were inferred by maximum likelihood, and the support obtained by this method was high and it strongly supported the monophyly of the genus *Ficus*. The phylogenetic tree shows that the genus *Ficus* is divided into five main branches (Figure 7). Cluster I contained eight species of the subgenus *Urostigma*. Seven species of the subgenus *Sycomorus* formed a monophyletic cluster with high support. Cluster III contained two species of the subgenus *Pharmacosycea* and *Ficus lyrata*. Cluster Ⅳ contained three species of the subgenus *Synoecia* and four species of the subgenus *Ficus*. Six species of the subgenus *Ficus* formed Cluster Ⅴ.

## 4. Discussion

### 4.1. Chloroplast Genomic DNA Structures

In the present study, we sequenced the eight *Ficus* species cp genomes, which laid the foundation for a comprehensive comparison of the cp genome sequences of Moraceae. The eight *Ficus* species reported in this study differ somewhat in chloroplast size, but in general they are roughly similar in chloroplast genome length to those reported so far for *Ficus* species, with all being around 160,000 bp in length [8,27]. The structure of the eight *Ficus* cp genomes, like most angiosperms, also has a typical quadripartite structure [8,45,46,47]. Moreover, we found that the content of the eight *Ficus* cp genomes was dominated by A/T, up to 64.1%, and this result was similar to that in other plants [48,49,50]. The lower G/C content of the *Ficus* genomes may be related to its own spontaneous mutations [51]. We also found that the GC content of LSC and SSC were lower than the IR regions, which were common in *Ficus* species [27,52,53], and this may be so due to the existence of rRNA and tRNA genes in the IR region [54].

### 4.2. Identification of SSRs and Repeat Sequences

Repeated sequences have been shown to be associated with the rearrangement, evolution and divergence of cp genome sequences, as well as having an important function in the phylogeny [12,55,56]. The number and distribution of the four repeats in the eight *Ficus* cp genomes, and the distribution of the repeats were similar in the cp genomes of *Ficus*, which was strongly related to the high conservation within the *Ficus* species. The cpSSR markers have been frequently used for intraspecific identification and genetic evolutionary analysis because of their high mutation rate [57,58,59]. The results of this study indicated that the mono-nucleotide repeats were the richer repeat type in the eight *Ficus* species, with the A/T being the enriched repeat motif, similar to other higher plant studies [13,60], indicating that the higher plants were dominated by low-level repeat motif [61]. The SSRs and repeats detected in this study were important for the subsequent phylogenetic studies and taxonomy of the genus *Ficus*, and even the family Moraceae. 

### 4.3. IR Constriction and Expansion

Scientists have noticed that the expansion and contraction of the IR regions affects the size of the chloroplasts [56,62]. Moreover, it may cause border genes to enter the IR or SC [63]. In our study, most of the genes (*rpl22*, *rpl2*, *ycf1*, *trnH*, and *psbA*) in the eight *Ficus* species were identical at the IR/SC boundary positions, and differed only in length, further suggesting that the *Ficus* species are closely related and have highly conserved cp genomes. The cp genomes of monocots and dicots have distinct differences in gene arrangement; for example, the *trnH* gene is located in the LSC region in dicots, whereas it is located in the IR region in monocots [64]. Interestingly, we found that the *rps19* gene of *F. auriculata* was located exclusively in the IRb region, which would be very rare in dicotyledons [65,66,67], and this may be the case because of expansion from the IR region to the LSC region.

### 4.4. Sequence Divergence Analysis

A comparative analysis of hypervariable regions in plant cp genomes can better identify mutation hotspots and provide a basis for genetic diversity analysis [68,69]. In this study, to identify variable regions, we calculated the percentage of variable characters (coding and non-coding regions) in eight *Ficus* species cp genomes. The results of the study were similar to those of angiosperms, where the non-coding regions were much more variable than the coding regions [70,71,72]. At the same time, we also found that the SC region of the eight *Ficus* species in this study were more variable than the IR region, which was consistent with previous results [73,74]. In previous studies, a large number of hypervariable regions were used as DNA barcodes for other plants [75,76,77]. We identified eight hypervariable regions in the *Ficus* cp genomes (*trnS-GCU_trnG-UCC*, *trnT-GGU_psbD*, *trnV-UAC_trnM-CAU*, *clpP_psbB*, *ndhF_trnL-UAG*, *trnL-UAG_ccsA*, *ndhD_psaC*, and *ycf1*). The discovery of these hypervariable regions could provide vast amounts of information for the development of molecular markers for phylogenetic analyses.

### 4.5. Non-Synonymous (Ka) and Synonymous (Ks) Substitution Rate Analysis

The Ka/Ks reveal selection pressure on protein-coding genes and they have a key role in evolutionary studies [78]. Non-synonymous nucleotide substitutions occurred with a lower frequency than synonymous substitutions in most genes, subject to purifying selection [65]. Thus, the ratio of Ka/Ks > 1 indicates probable positive selection; Ka/Ks < 1 indicates purifying selection, while Ka/Ks values of nearly 1 indicate neutral evolution [79]. The results of this study showed that a great many genes had Ka and Ks values that are equal to 0, indicating that these eight *Ficus* chloroplast genomes are relatively conservative. However, the highest Ka and Ks values were for the gene between *F. altissima* and *F. religiosa* (ka = 2.6149, ks = 5.0893), suggesting that it is more variable in *F. altissima*. The ycf1 gene was under positive selection in the *Ficus* cp genome, and so the ycf1 gene may have an important role in the adaptation of *Ficus* species to different environments [80]. However, it cannot be excluded that it may be a pseudogene, as the gene often becomes pseudogenic in angiosperm cp genomes [81]. Therefore, further studies are needed to determine whether the ycf1 gene is in a positive selection state in the *Ficus* cp genome. The Ka/Ks values were less than 1 for all genes except *ycf1*, which indicated that these cp genomes underwent extensive purifying selection.

### 4.6. Phylogenomic Analysis

The cp genome was the most vital genetic resource for inferring plant evolutionary relationships and was well suited for analyzing comparative plant relatedness [82,83]. In this study, in order to determine the phylogenetic relationships of eight *Ficus* genus, phylogenetic trees were structured using ML methods. The findings indicated that all *Ficus* species clustered in the one clade with high values, a finding that is consistent with the phylogeny inferred by previous work using cpDNA fragments [8]. 

According to the results of the phylogenetic analysis, species of the three subgenera (subgenus *Sycomorus*, subgenus *Synoecia*, and subgenus *Pharmacosycea*) were all monophyletic taxa and had strong support values, suggesting that the cp genome was suited for resolving the phylogeny of the *Ficus*. Berg [4] has pointed out the existence of some morphological similarities between the subgenus *Ficus* and the subgenus *Synoecia*. The results of the present study showed that the subgenus *Ficus* and the subgenus *Synoecia* are clustered into one branch, indicating a close relationship between the two subgenera. Therefore, the taxonomic treatment of the subgenus *Synoecia* and subgenus *Ficus* requires further study. 

The results of Rønsted [84] suggest that the subgenus *Urostigma* belongs to a non-monophyletic origin, and we obtained similar results. Interestingly, in the present study, *Ficus lyrata* showed a close relationship with the subgenus *Pharmacosycea*, and so further research is needed on the subgenus classification of *F. lyrata*. 

Based on morphological traits [85] as well as molecular data [6], it was previously suggested that *Ficus tikoua* should be classified as subgenus *Sycomorus* and our phylogenetic analysis provides strong evidence for this classification. Subgenus *Urostigma* and subgenus *Sycomorus* formed a clade, indicating that the two subgenera are closely related. Zhang [86] points out that *F. auriculata* and *F. beipeiensis* should be classified as the same species, but our study results clearly do not support this classification. In our result, *F. auriculata* and *F. beipeiensis* were still somewhat different, despite being more closely related. Our results show that the *F. formosana*, *F. heteromorpha*, *F. erecta* and *F. pandurate* of the *Ficus gasparriniana*-*F. heteromorpha* complex of sect. *Ficus* cluster together, a result that is consistent with those obtained in previous phylogenetic studies based on SSR markers [87].

## 5. Conclusions

In our study, we assembled and analyzed the cp genomes of eight *Ficus* species. By comparing these cp genomes, the results show that the structures of these cp genomes are similar to those of most angiosperm genomes, which exhibit a typical tetragonal structure. Additionally, we obtained valuable genetic resources, including SSRs, highly variable loci, and repetitive sequences. Phylogenetic analysis shows that all *Ficus* species were clustered in the same clade. The present study provides a clearer phylogenetic framework for the taxonomy of the genus *Ficus*, and it will help in the species identification of these species, and in genetic diversity studies.

## Figures and Tables

**Table 1:** List of genes encoded by eight species of *Ficus* chloroplast genome. (×2) indicates that the gene has two copies. * and ** indicate genes containing one/two introns.

Category	Gene Groups	Gene Names	Number
Self-replication	Large subunit of ribosomal proteins	rpl14, rpl16 *, rpl2 (×2) *, rpl20, rpl22, rpl23 (×2), rpl32, rpl33, rpl36	11
	Small subunit of ribosomal proteins	rps11, rps12 **, rps14, rps15, rps16 *, rps18, rps19, rps2, rps3, rps4, rps7 (×2), rps8	13
	DNA dependent RNA polymerase	rpoA, rpoB, rpoC1 *, rpoC2	4
	ribosomal RNAs	rrn16 (×2), rrn23 (×2), rrn4.5 (×2), rrn5, (×2)	8
	transfer RNAs	trnA-UGC (×2) *, trnC-GCA, trnD-GUC, trnE-UUC, trnF-GAA, trnG-GCC, trnG-UCC *, trnH-GUG, trnI-CAU (×2), trnI-GAU (×2) *, trnK-UUU *, trnL-CAA (×2), trnL-UAA *, trnL-UAG, trnM-CAU, trnN-GUU (×2), trnP-UGG, trnQ-UUG, trnR-ACG (×2), trnR-UCU, trnS-GCU, trnS-GGA, trnS-UGA, trnT-GGU, trnT-UGU, trnV-GAC, (×2), trnV-UAC *, trnW-CCA, trnY-GUA	36
Photosynthesis	Photosystem I	psaA, psaB, psaC, psaI, psaJ	5
	Photosystem II	psbA, psbB, psbC, psbD, psbE, psbF, psbH, psbI, psbJ, psbK, psbM, psbN, psbT, psbZ	14
	NADP dehydrogenase	ndhA *, ndhB (×2) *, ndhC, ndhD, ndhE, ndhF, ndhG, ndhH, ndhI, ndhJ, ndhK	12
	Cytochrome b6/f complex	petA, petB *, petD *, petG, petL, petN	6
	ATP synthase	atpA, atpB, atpE, atpF *, atpH, atpI	6
	Rubisco	rbcL	1
Other genes	Maturase	matK	1
	Protease	clpP **	1
	Envelop membrane protein	cemA	1
	Subunit Acetyl- CoA-Carboxylate	accD	1
	c-type cytochrome synthesis gene	ccsA	1
Unknown	Conserved Open reading frames	*ycf1* (×2), ycf2, (×2), ycf3 **, ycf4	6
			127

## Data Availability

The chloroplast genomes sequences of eight *Ficus* species (*F. altissima*, *F. auriculata*, *F. benjamina*, *F. curtipes*, *F. heteromorpha*, *F. lyrata*, *F. microcarpa*, *F. virens*) have been uploaded to NCBI and the GenBank accession numbers are MW043478.1, MW039147.1, MW039146.1, MW039143.1, MW039145.1, MW039148.1, MW039144.1, MW039142.1.

## Supplementary Materials

The following supporting information can be downloaded at: https://www.mdpi.com/article/10.3390/life12060848/s1. Table S1: List of samples voucher collection information and GenBank accessions; Table S2: The 34 chloroplast genomes used for phylogenetic analysis; Table S3: Base composition in the chloroplast genomes of *Ficus* species; Table S4: Types and numbers of SSRs motifs in eight *Ficus* cp genomes; Table S5: Repeats in the chloroplast genome of eight *Ficus* species; Table S6 The rates of Ka, Ks and Ka/Ks of 53 protein coding genes in 8 *Ficus* species by pairwise comparison computed by KaKs_Calculator with default parameters.
